# Coding-complete genome sequence and phylogenetic analysis of Coxsackievirus B3 isolated from South Korea

**DOI:** 10.1128/mra.01160-23

**Published:** 2024-05-23

**Authors:** Yun Ji Ga, Yun Young Go, Jung-Yong Yeh

**Affiliations:** 1 Department of Life Sciences, College of Life Sciences and Bioengineering, Incheon National University, Yeonsu-gu, Incheon, South Korea; 2 College of Veterinary Medicine, Konkuk University, Gwangjin-gu, Seoul, South Korea; Portland State University, Portland, Oregon, USA

**Keywords:** Coxsackievirus B3, genome, *human enterovirus*, phylogenetic analysis

## Abstract

Whole-genome sequencing of a Coxsackievirus B3 strain isolated from the stool of a febrile patient with aseptic meningoencephalitis, South Korea, in 2002 was performed. This strain exhibits a high nucleotide sequence identity with various strains circulating in China from 2001 to 2019.

## ANNOUNCEMENT

Coxsackievirus B3 (CVB3), a member of the *human enterovirus B* species, belongs to the genus *Enterovirus* in the family *Picornaviridae* (1). CVB3 is mainly associated with aseptic meningitis and pericarditis; there have been reported cases of global transmission causing pancreatic and gastrointestinal disorders (2–6), acute flaccid paralysis (7), and more recently, hand, foot, and mouth disease (8–10). Despite its significance, to our knowledge, no genetic analysis of the whole-genome sequence of CVB3 strains isolated in South Korea has been reported (Table 1). Thus, this study aims to bridge this gap by conducting a thorough genomic analysis, determining the coding-complete genome sequence of a CVB3 strain circulating in the country. Here, we present a coding-complete genome sequence of a CVB3 strain isolated in South Korea, enhancing our understanding of the genetic relatedness of CVB3 in the Asia-Pacific region.

**TABLE 1:** Identity analysis of partial nucleotide sequences in Korean Coxsackievirus B3 strains registered in the GenBank database

Coxsackievirus B3 strain	Region (nt)	GenBank accession number	Identity
Kor10-CVB3-583cn	VP1 (321 nt)	KX256191.1	290/321 (90%)
Kor10-CVB3-522cn	VP1 (321 nt)	KX256190.1	290/321 (90%)
CB3/2005/Seoul-16	VP1 (268 nt)	DQ530390.1	239/268 (88%)
p19	5′UTR (137 nt)	DQ167421.1	127/137 (90%)
p18	5′UTR (137 nt)	DQ167420.1	127/137 (90%)
p10	5′UTR (137 nt)	DQ167416.1	128/137 (90%)
p6	5′UTR (137 nt)	DQ167412.1	128/137 (90%)
CB3/2005/Seoul-16	5′UTR (563 nt)	DQ530412.1	522/563 (91%)

CVB3 KR-2002 strain isolated from the stool of a febrile patient with aseptic meningoencephalitis in Jeollabuk-do, South Korea, in 2002 was acquired from the National Culture Collection for Pathogens (Osong-eup, Cheongju-si, South Korea). The virus was serially passed five times in Vero cells, and the virus was harvested from the infected cell cultures before RNA extraction using QIAamp Viral RNA Mini Kit (Qiagen, Valencia, CA, USA). Sequencing libraries were prepared using the Illumina Stranded Total RNA prep ligation with Ribo Zero Plus Library prep kit (Illumina, San Diego, CA, USA) and sequenced on an Illumina Miseq 300 bp paired-end reads. The quality control of the 8,576,486 reads was assessed using FastQC v0.11.9, and the adapters were trimmed using Trimmomatic v0.39 with the following parameters: ILLUMINACLIP:2:30:10:2:keepBothReads LEADING:10 TRAILING:10 MINLEN:100 (11, 12). The CVB3 whole genomes were then *de novo* assembled using SPAdes 3.15.2 (13). All tools were run with default parameters unless otherwise specified.

**Fig 1:**
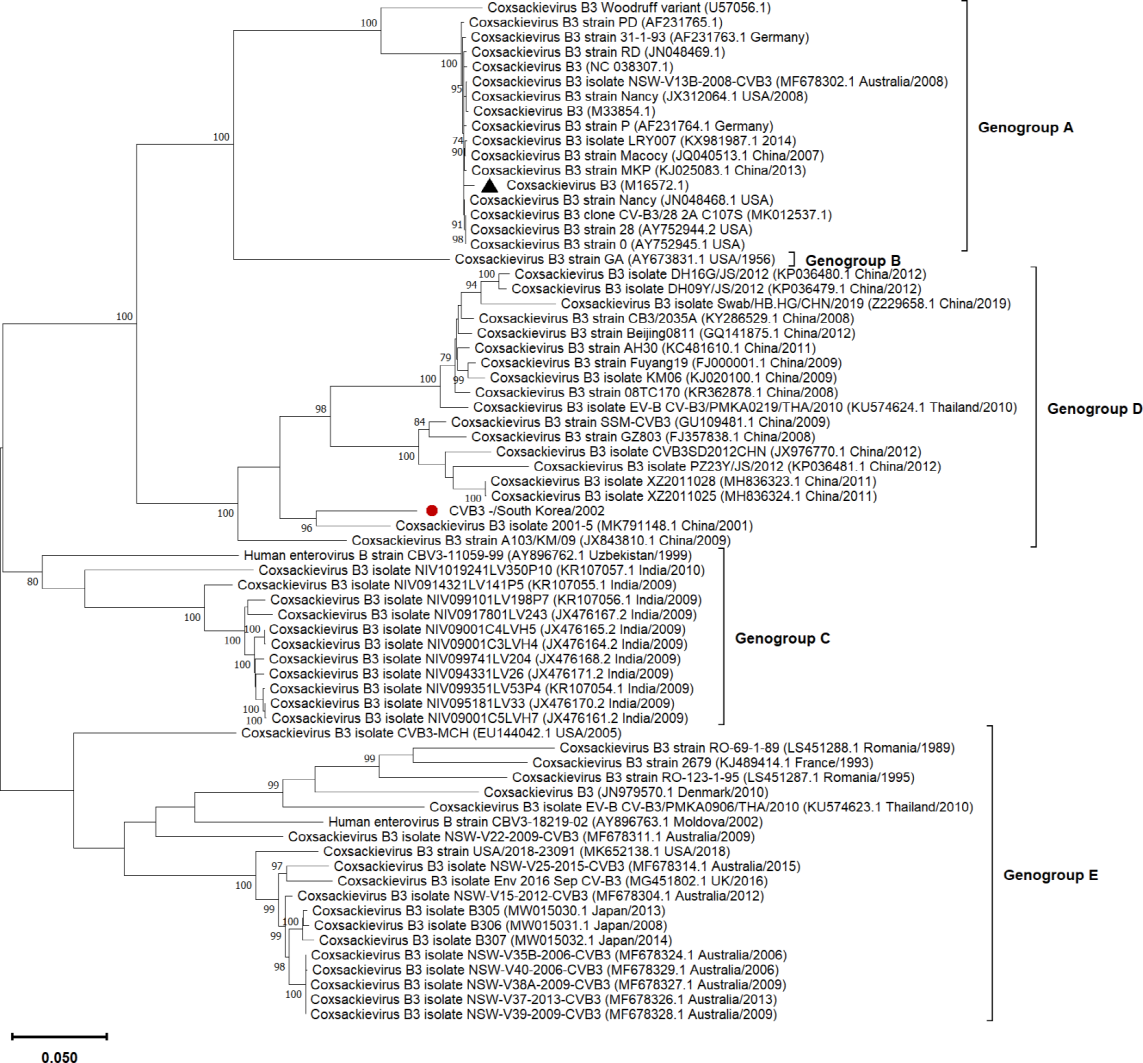
Phylogenetic tree based on the coding-complete genomes of 69 Coxsackievirus B3 (CVB3) strains from around the world. The tree was constructed using the maximum-likelihood method (based on the Tamura-Nei model) with bootstrap values calculated from 1,000 replicates using the MEGA program (version 11.0). The entire open reading frame sequence (6,555 nt length) was used for the analysis using the BioEdit sequence alignment editor software. The GenBank accession numbers and, if available, the country and year of isolation are shown for the other reference strains. The CVB3 strains were categorized into five Genogroups (A–E). In the phylogenetic tree, the CVB3 KR-2002 strain is denoted by a red filled circle, and the prototype strain isolated in 1987 is indicated by a filled triangle. Bootstrap values above 70% are displayed above the branches, indicating the statistical support for the clustering. The scale bar represents the genetic distance between the strains in the tree.

The coding-complete genome of the CVB3 KR-2002 strain was found to contain 7,386 nucleotides (nt), excluding the poly (A) tail. The 5′untranslated region (UTR) was found to be 731 nt, followed by a single open reading frame (ORF) encoding a large polyprotein (2,186 amino acids), and the 3′ UTR was 97 nt long. The CVB3 KR-2002 strain genome consists of 28.12% A, 22.98% C, 24.51% G, and 24.4% U with a G + C content of 47.49%. Phylogenetic analysis using the maximum likelihood method based on the coding-complete genome reveals that KR-2002 CVB3 strain belongs to the genogroup D (Fig. 1) (10). The CVB3 KR-2002 strain is more closely related to CVB3 2001-5 strain from China of genogroup D (GenBank accession no. MK791148.1; 91% nucleotide identity in ORF). As shown in Fig. 1, genogroup D consists almost exclusively of strains isolated in China, with the exception of one isolate from Thailand in 2010 (GenBank accession no. KU574624.1). From 1990 to 2010, the majority of strains isolated in Guangdong, Jilin, Anhui, Yunnan, and Beijing (14) provinces of China belonged to genogroup D. Notably, genogroup D contains the CVB3 strain responsible for the 2008 outbreak of aseptic meningitis in Shandong Province, China (15).

This study identifies the coding-complete genome sequence of a CVB3 strain isolated in South Korea. The CVB3 strain KR-2002, identified in South Korea in 2002, exhibits a high nucleotide sequence identity with various strains circulating in China from 2001 to 2019. This suggests a possible connection between CVB3 strains in both regions.

## Data Availability

The full-length genome sequence of the CVB3 strain KR-2002 isolated in Jeollabuk-do, South Korea, in 2002 has been deposited in GenBank under accession no. OQ919474.1. The Illumina MiSeq sequence raw reads in the NCBI Sequence Read Archive (SRA) are available at https://www.ncbi.nlm.nih.gov/sra/SRX23633849[accn]; the BioProject number is PRJNA1076842.
